# Homotypic cell membrane-cloaked biomimetic nanocarrier for the accurate photothermal-chemotherapy treatment of recurrent hepatocellular carcinoma

**DOI:** 10.1186/s12951-020-00617-2

**Published:** 2020-04-16

**Authors:** Yingxue Sun, Wenhui Zhai, Xiaojun Liu, Xiangyi Song, Xiaonan Gao, Kehua Xu, Bo Tang

**Affiliations:** 1 College of Chemistry, Chemical Engineering and Materials Science, Key Laboratory of Molecular and Nano Probes, Ministry of Education, Collaborative Innovation Center of Functionalized Probes for Chemical Imaging in Universities of Shandong, Institute of Molecular and Nano Science, Shandong Normal University, Jinan, 250014 People’s Republic of China; 2 College of Geography and Environment, Shandong Normal University, Jinan, 250014 People’s Republic of China

**Keywords:** Homotypic cell membrane, Photothermal therapy, Chemotherapy, Synergistic therapy, Recurrent hepatocellular carcinoma

## Abstract

**Background:**

Tumor recurrence in patients after surgery severely reduces the survival rate of surgical patients. Targeting and killing recurrent tumor cells and tissues is extremely important for the cancer treatment.

**Results:**

Herein, we designed a nano-biomimetic photothermal-controlled drug-loading platform **HepM-TSL** with good targeting ability and immunocompatibility for the treatment of recurrent hepatocellular carcinoma. **HepM-TSL** can accurately target the recurrent tumor area with the aid of the cloaked homotypic cell membrane and release the chemotherapy drugs in a controlled manner. In vivo results have confirmed that **HepM-TSL** loaded with drugs and photosensitizer achieves the synergistic treatment of recurrent hepatocellular carcinoma with good therapeutic effect and slight side effects.

**Conclusion:**

Accordingly, **HepM-TSL** provides a sound photothermal-chemotherapy synergistic strategy for the treatment of other recurrent cancers besides of recurrent hepatocellular carcinoma.

## Background

Hepatocellular carcinoma (HCC), as the third leading cause of cancer-related mortality worldwide, is a common malignant tumor that seriously endangers human health [1–3]. HCC was diagnosed till advanced stages for lacking of effective therapies [4–6]. At present, partial hepatectomy is a relative curative treatment preferentially for primary HCC patients, which can effectively treat tumor and improve the survival rate of patients [2, 7–9]. Regrettably, 70–80% of patients undergo tumor recurrence within 5 years after surgery, greatly reducing the survival rate after surgery [10]. The high recurrence rate of HCC is an important issue in the treatment of liver cancer. As a result, the treatment of recurrent HCC is an urgent problem to be solved. So far, there are no current consensus guidelines to treat the patients with recurrent HCC. The current treatment methods mainly include repeat hepatectomy (RH), radiofrequency ablation (RFA) or transarterial chemoembolization (TACE) [11–13]. In theory, the best way to treat recurrent HCC is repeated hepatectomies and liver transplantation. However, due to the practical obstacles including multicentric tumors, extrahepatic spread and inadequate normal liver reserve, repeated hepatectomies are available only for selected patients. TACE and RFA may lead to small survival benefits [14–17]. In general, the therapeutic efficacy of single therapy is still dismal.

The combination of photothermal-chemotherapy treatment brings effective therapy due to the synergistic effect [18, 19]. Even so, problems include low targeting and low delivery efficiency of drug and photosensitizer still exist in current combination therapy [20–24]. Therefore, it is significant to design a nano-drug delivery platform that has good delivery and controlled-release effects for chemotherapy drugs and photothermal agents as well as precise target to tumor area. To this point, the use of the homotypic cancer cell membrane as the cloak of nano-drug delivery platform would be one effective strategy [25–31]. Once the nano-drug delivery platform is enveloped with the homotypic cancer cell membrane, the loaded chemotherapy drugs and photothermal agents will be released controllably in the tumor area due to the homotypic targeting ability of the cancer cell membrane, so as to improve the synergistic efficacy of photothermal therapy and chemotherapy.

**Scheme 1:**
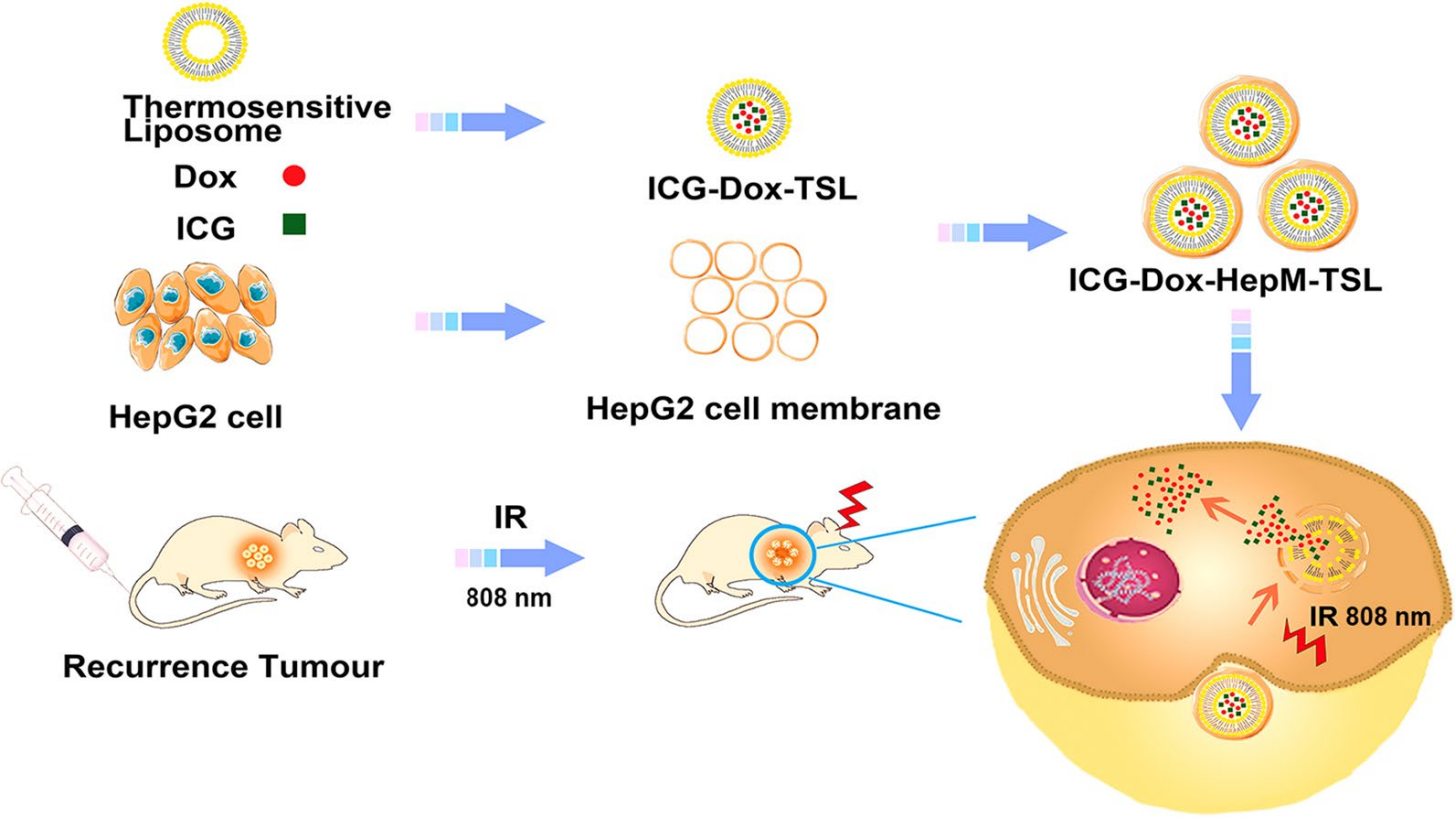
The design strategy of the nano-drug delivery platform ICG-Dox-**HepM-TSL**

Herein, in order to effectively treat the recurrent HCC through the combination of photothermal therapy and chemotherapy, we designed a drug delivery platform using homotypic cancer cell membrane as the cloak and realized the synergistic treatment of recurrent HCC with good therapeutic effect and negligible side effects. As shown in Scheme 1, the nano-drug delivery platform **HepM-TSL** constructed by the thermosensitive liposome (TSL) vesicles which were coated with the HCC cell membrane, and the chemotherapy drug (doxorubicin, Dox) and photosensitizer (indocyanine green, ICG) were encapsulated into **HepM-TSL**, noted as ICG-Dox-**HepM-TSL**. With the help of the homotypic HCC cell membrane, ICG-Dox-**HepM-TSL** can escape the immune system and precisely target the recurrent HCC area. The encapsulated DOX and ICG could be controllably released in tumor area when decomposition of TSL was induced by laser irradiation, and photothermal-chemo synergistic therapy was achieved. The in vivo results proved that the recurrent HCC in the mice drastically reduced with the therapy of ICG-Dox-**HepM-TSL**, accompanied by slight side effects on the normal organs and tissues. In a word, nano drug delivery platform developed in this work can target the tumor area precisely, and release the drug in a controlled manner, making the combination of photothermal and chemotherapy effective, which is expected to provide a basis for the treatment of recurrent tumors.

## Methods

### Preparation of TSL nanoparticles

The blank heat-sensitive liposome nanoparticles were placed in dialysis bag (3500D) and dialyzed in secondary water. After 8 h, the pH value of the internal and external aqueous phase was adjusted to make the pH value of the external aqueous phase 7.8. Then, the chemotherapeutic drug Dox and the photosensitizer ICG and the thermosensitive liposome nanoparticles were added to the liposome solution at a ratio of 1:1:10, and mixed evenly, then the above mixed solution were incubated in a 39 °C water bath for 30 min. The mixture was then placed in a dialysis bag for 24 h to dialyze off the free chemotherapeutic drug and photosensitizer.

### Preparation of cell membrane-cloaked TSL nanoparticles

2 mL of TSL nanoparticles (1.0 mg/mL) was mixed with 1 mL of HepG2 cell or L02 cell membrane vesicles (0.5 mg/mL). Then, the mixture was sonicated for 10 min (40 kW). Then, the cell membrane-cloaked TSL nanoparticles were collected by centrifugation treatment at 14,000 rpm for 10 min at 4 °C, and the supernatant was discarded. The cell membrane-cloaked TSL nanoparticles were resuspended in 3 mL of secondary water. The concentration of Dox in the stock liquid was 41.32 μg/mL.

### Flow cytometry analysis

Flow cytometry was used to assess the in vitro therapeutic effect of ICG-Dox-**HepM-TSL**. HepG2 cells were seeded and cultured for 24 h in 2 mL of DMEM (10% FBS). After the supernatant was discarded, the HepG2 cells were incubated with ICG-Dox-**HepM-TSL** and ICG-Dox-TSL and free Free-ICG-Dox for 4 h. The incubation buffer was prepared by diluting the stock solution, 24.2 μL of ICG-Dox-HepM-TSL/ICG-Dox-TSL was mixed with 975.8 μL of DMEM containing 10% FBS, and finally the concentration of Dox in the final incubation solution was 5 μg/mL. The concentration of Dox in ICG-Dox-HepM-TSL, ICG-Dox-TSL nanoparticles, free-ICG-DOX was kept the same. The above cells were divided into two groups, one of which was irradiated with infrared light and the other group was used as a blank control. After the incubation buffer was discarded, the cells were trypsinized (free from EDTA), collected by centrifugation at 800 rpm for 5 min and washed thrice with PBS (pH 7.4). Finally, the Annexin V-FITC/PI Apoptosis Detection Kit were used to stain the above cells and an Image-StreamX multispectral imaging flow cytometer (Amnis Corporation) was used to examine the apoptosis of these cells. The flow cytometry data were analyzed using IDEAS software.

### In vivo tumor image

Select 4 to 6 weeks of BALB/c nude mice weighing 15–20 g were used. The mice were housed in cages (5 per cage) and regularly fed rat chow and water. In order to build a solid tumor of HCC in nude mice subcutaneously, 5 × 10^6^ HepG2 cells were injected subcutaneously into the flank region of the nude mice. When the tumor volume of the nude mice reached 100–200 mm^3^, Surgical removal of tumors from mice and retention of 10 mm^3^ tumor tissue for simulated tumor recurrence. After 1 week of recovery, the mice were randomly divided into 3 groups and the tail intravenous injected with ICG-Dox-HepM-TSL and its counterparts at regular intervals. The quality of the drug was the same in each group (5 mg/kg mouse). The concentration of Dox in ICG-Dox-HepM-TSL, ICG-Dox-TSL nanoparticles were kept same. Group 1 was injected with 100 μL of PBS with or without near-infrared irradiation and was the control group, Group 2 was injected with ICG-Dox-TSL solution with or without near-infrared irradiation, and Group 3 was injected with ICG-Dox-**HepM-TSL** solution with or without near-infrared irradiation. 24 h after mice tail intravenous injection,they were irradiated with NIR for 5 min.

After the above groups of mice were treated for 13 days, the nude mice were subjected to the live imager and photothermal imager and the fluorescence imaging of Dox in the nude mice were collected. Then, 24 h after the final injection the nude mice were sacrificed and the blood were collected by cardiac puncture. Dox fluorescence imaging was performed for ex vivo tissue from the main organs (heart, liver, spleen, lung and kidney) and tumors. H&E staining was performed on the main organs and tumor tissues. Then the collected blood was centrifuged at 3000 rpm for 10 min and the serum were collected. The serum was dripped into the 96-well plate, then follow the instructions to measure the absorbance of the alkaline phosphatase (ALP), alanine aminotransferase (ALT), aspartate aminotransferase (AST), blood urea nitrogen (BUN) and serum creatinine (Cre) with an ELISA microplate reader. The experiment was repeated three times, and the data are shown as the mean ± SD. The body weights and tumor volumes of the mice were measured during treatment. All animal experiments were carried out according to the Principles of Laboratory Animal Care (People’s Republic of China) and the Guidelines of the Animal Investigation Committee, Biology Institute of Shandong Academy of Science, China. The statistical data were analyzed using SPSS Statistics software, for deriving standard deviation, one-way ANOVA test and Bonferroni test. A p value of 0.05 was taken as the level of significance and the data were labeled with (*) for P < 0.05, and for (**) for P < 0.01, Each experiment was conducted in triplicate (n = 3).

### Hemolysis assay

The blood from BALB/c mice were centrifuged at 4000 rpm for 5 min and the supernatant was discarded. The erythrocytes cells were washed with PBS three times until the supernatant became clear and transparent. Finally, the erythrocytes cells were resuspended in PBS and diluted to 2 v/v%. The above erythrocytes cells were respectively mixed with different concentrations of ICG-Dox-HepM-TSL, ICG-Dox-TSL, ICG, Dox, and Tween-80 for 4 h at 37 °C. After 4 h, the mixtures were centrifuged at 1500 rpm for 15 min, and then the supernatant was collected and its absorbance (A_sample_) was measured at 540 nm with an ELISA microplate reader. Erythrocytes were incubated with PBS as the positive control (A_100_) and deionized water as the negative control (A_0_). The hemolysis rate of the experimental group was calculated as follow. The experiment was repeated three times, and the data are shown as the mean ± SD.

$${\text{Hemolysis rate }}\left( \% \right)\, = \,\left( {{\text{A}}_{\text{sample}} {-}{\text{A}}_{0} } \right)/\left( {{\text{A}}_{ 100} {-}{\text{A}}_{0} } \right)\, \times \, 100.$$

### Statistical analysis

All the statistical data were analyzed using SPSS Statistics software, for deriving standard deviation, one-way ANOVA test and Bonferroni test. A p-value of 0.05 was taken as the level of significance and the data were labeled with (*) for P < 0.05, and for (**) for P < 0.01, Each experiment was conducted in triplicate (n = 3).

## Results and discussion

### Preparation and characterization of **HepM-TSL**

**Fig. 1:**
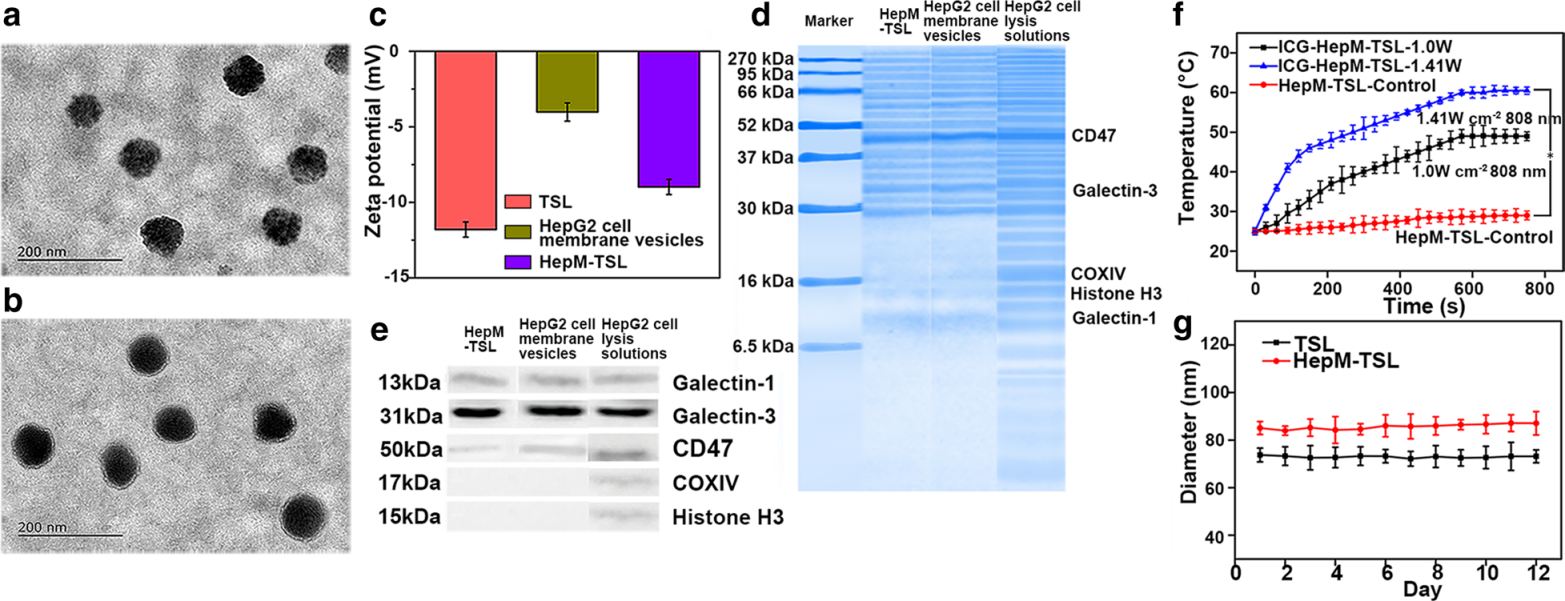
Preparation and characterization of **HepM-TSL**. **a** TEM image of bare TSL nanoparticles. **b** TEM image of **HepM-TSL** nanoparticles. **c** Zeta potentials of bare TSL nanoparticles, **HepM-TSL** nanoparticles and HepG2 cell membrane vesicles. **d** Gel electrophoresis analysis of **HepM-TSL** nanoparticles, HepG2 cell membrane vesicles and HepG2 cell lysis solutions. **e** Western blot analysis of **HepM-TSL** nanoparticles, HepG2 cell membrane vesicles and HepG2 cell lysis solutions. Samples were run with equal protein concentrations and immunostained against membrane markers, including galectin-1, CD47 and galectin-3, and intracellular markers, including histone H3 (a nuclear marker), cytochrome c oxidase (COXIV, a mitochondrial marker). **f** The photothermal responses of the **HepM-TSL** nanoparticles with ICG concentration of 50 μg mL^−1^ under irradiation with an NIR laser (808 nm, 1.0 W cm^−2^ and 1.41 W cm^−2^). **g** Stability of TSL nanoparticles and **HepM-TSL** nanoparticles in PBS. The TSL nanoparticles and **HepM-TSL** nanoparticles were dispersed in PBS at room temperature then kept for 12 days. The diameters of the nanoparticles were measured with a dynamic light scattering (DLS) analyzer every day

TSL nanoparticles were first synthesized and the **HepM-TSL** nanoparticles were prepared with HepG2 cell membranes as the cloak. Cell membranes were obtained from the HepG2 cells according to previous literatures [32, 33]. TSL nanoparticles, **HepM-TSL** nanoparticles and HepG2 cell membrane were characterized with varied approaches. (Figure 1a, b and Additional file 1: Fig. S1). The Zeta potential the **HepM-TSL** changes much compared to TSL (Fig. 1c). The protein ingredient analysis of **HepM-TSL** was verified with gel electrophoresis (Fig. 1d), and the membrane proteins profile of **HepM-TSL** were similar to those of HepG2 cell membrane vesicles, ensuring the intact retain of the HepG2 cell membrane during the preparation procedure. The western blot (WB) analysis results (Fig. 1e) illustrated that the main cellular adhesion molecules including galectin-1, galectin-3 and CD47 were enriched on the surface of **HepM-TSL** while the main intracellular nuclear marker and mitochondrial marker i.e. histone H3 and COXIV were little on the HepG2 cell membrane. Results in Fig. 1d, e confirmed the selective retention of membrane on the surface of **HepM-TSL**.

The photothermal effects of the **HepM-TSL** with ICG were investigated by measuring the elevated temperatures of their suspensions (50 μg mL^−1^) under 808 nm NIR laser irradiation (1 W cm^−2^, 1.41 W cm^−2^, 720 s). As the power increased, the final temperature of the **HepM-TSL** loaded with ICG was elevated to near 60 °C (the highest final temperature) (Fig. 1f), reaching the temperature requirement for heat killing of the tumor, which means that the TSL with ICG can efficiently convert NIR laser into heat. The stabilities of **HepM-TSL** and the TSL nanoparticles were measured using a dynamic light scattering (DLS) analyser (Fig. 1g). After 12 days, the particle size of **HepM-TSL** hardly changed. In summary, it was proved that the HepG2 cell membrane coated with thermosensitive liposome was prepared with excellent stability and photothermal performance.

### Validating the homologous targeting ability of **HepM-TSL**

**Fig. 2:**
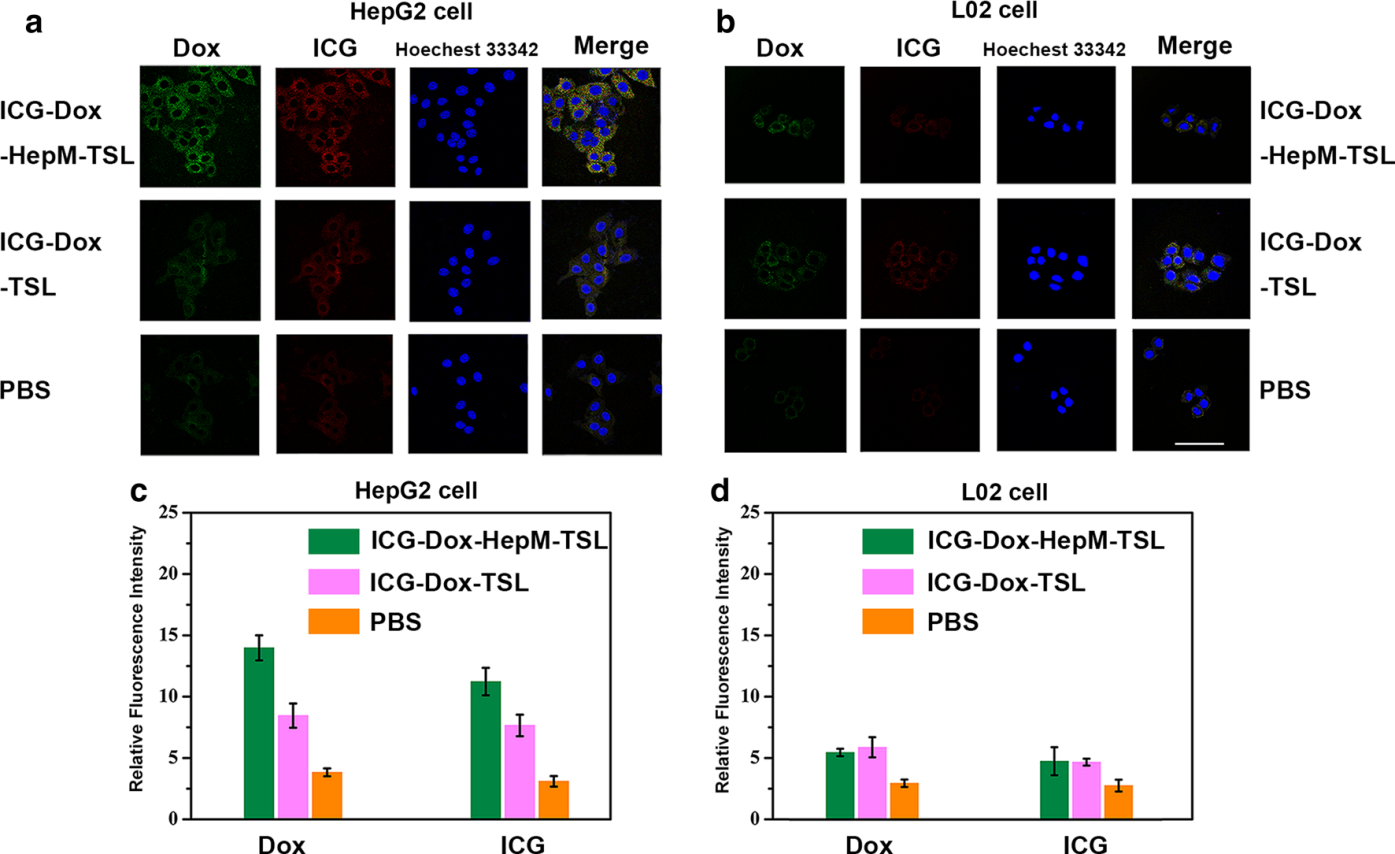
Validating the homologous targeting ability of **HepM-TSL**. Fluorescence images of HepG2 cells (**a**) and L02 cells (**b**) incubated with ICG-Dox-**HepM-TSL**, ICG-Dox-TSL nanoparticles and PBS for 4 h. **c** Quantitative histograms of the Dox fluorescence intensities and ICG fluorescence intensities in (**a**). **d** Quantitative histograms of the Dox fluorescence intensities and ICG fluorescence intensities in (**b**) Scale bar: 75 μm. The concentration of Dox in ICG-Dox-**HepM-TSL**, ICG-Dox-TSL nanoparticles, free-ICG-DOX were kept the same

The targeting ability of tumor cells by ICG-Dox-**HepM-TSL** relies on the ability of homotypic aggregation between homologous tumor cells. The study of targeting ICG-Dox-**HepM-TSL** to tumor cells was carried out and results were shown in Fig. 2. Hepatoma cells (HepG2 cells) and normal hepatocytes (L02 cells) were incubated with ICG-Dox-**HepM-TSL**, ICG-Dox-TSL nanoparticles, PBS, respectively for 4 h and then characterized with CLSM. The fluorescence intensity of HepG2 cells incubated with ICG-Dox-**HepM-TSL** was significantly stronger than that of the cells treated with ICG-Dox-TSL (Fig. 2a–c). As to L02 cells, the fluorescence intensities show little difference in the cells incubated with ICG-Dox-**HepM-TSL** and ICG-Dox-TSL (Fig. 2b–d). The ability to homotypic aggregation of ICG-Dox-**HepM-TSL** was verified by further experiments as shown in Additional file 1: Fig. S2 and S3. HepG2 cell, BGC-823 cell, Hela cell and MCF-7 cell were incubated with ICG-Dox-**HepM-TSL** for 4 h and then examined by the flow cytometric assay and CLSM. It was showed that ICG-Dox-HepM-TSL precisely targeted to HepG2 cells instead of other cancer cells. The above results indicate that ICG-Dox-**HepM-TSL** can target to HepG2 cells by virtue of the ability of homologous aggregation of HCC cell membranes and can target to recurrent HCC tumor regions.

### Drug release and MTT assay

**Fig. 3:**
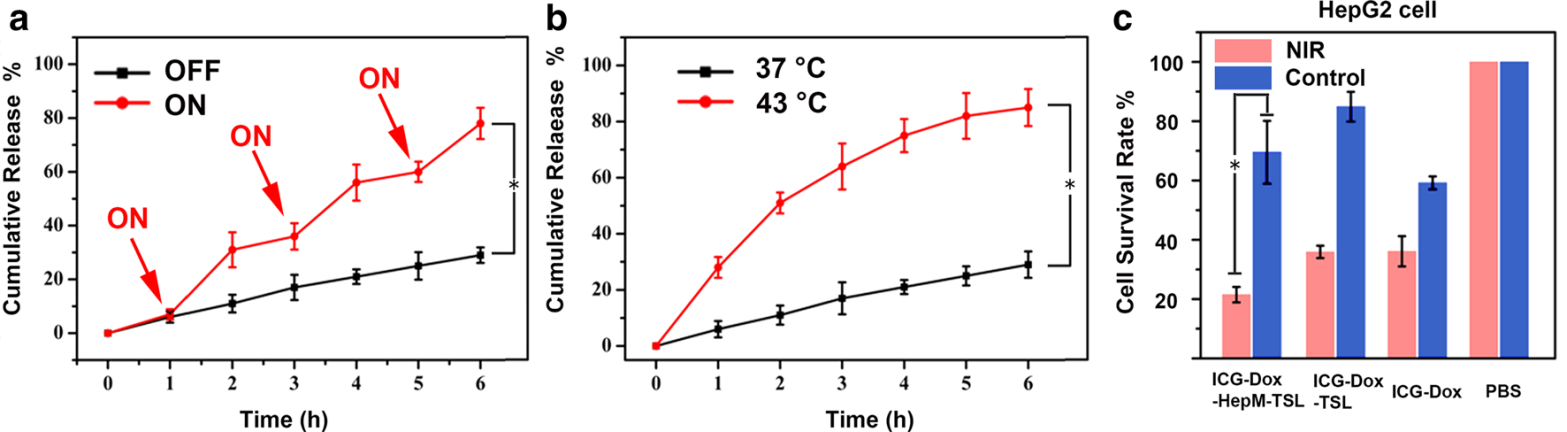
Drug release and MTT assay. **a** The in vitro release profiles of Dox from ICG-Dox-**HepM-TSL** with or without of near-infrared irradiation at 37 °C. **b** The in vitro release profiles of Dox from ICG-Dox-**HepM-TSL** at 37 °C and 43 °C without of near-infrared irradiation. **c** MTT assay results of ICG-Dox-**HepM-TSL** toward HepG2 cell. The concentration of Dox in ICG-Dox-**HepM-TSL**, ICG-Dox-TSL nanoparticles, free-ICG-DOX were kept the same

As one drug carrier platform, the drug loading capacity and the cumulative drug release efficiency of ICG-Dox-**HepM-TSL** in the tumor area are important issues for the treatment of recurrent tumors. Herein, Dox and ICG loading contents in ICG-Dox-**HepM-TSL** were determined with standard curves (Additional file 1: Fig. S4) and were 41.32 μg/mg and 34.83 μg/mg, respectively. Afterwards, the in vitro cumulative release profiles of Dox from ICG-Dox-**HepM-TSL** treated with laser were investigated. Under the irradiation of near-infrared laser, ICG converted laser into large amount of heat, making the thermosensitive liposome broken and Dox release. The cumulative drug release Dox from ICG-Dox-**HepM-TSL** in the presence or absence of near-infrared laser (808 nm) were studied (Fig. 3a, b). Results authenticated that, after three times irradiation, the cumulative release amount of DOX reached 81%, largely higher than that of the control group without laser irradiation (24%). As can be seen, the thermosensitive liposome in ICG-Dox-**HepM-TSL** made Dox controlled release by the NIR laser. The therapeutic effect of ICG-Dox-**HepM-TSL** was evaluated by the MTT assay with HepG2 cells (Fig. 3c) Cell viability of HepG2 cells treated with ICG-Dox-**HepM-TSL** was almost 70% in the absence of NIR irradiation, but sharply decreased as to 20% after NIR irradiation. Meanwhile, under irradiation of NIR, cells treated with ICG-Dox-**HepM-TSL** showed much lower viability compared with those treated with ICG-Dox-TSL or free ICG-Dox. These results verified that ICG-Dox-**HepM-TSL** had excellent photothermal-chemotherapy therapy efficiency against the recurrent HCC tumor cells.

### In vitro therapeutic effect

**Fig. 4:**
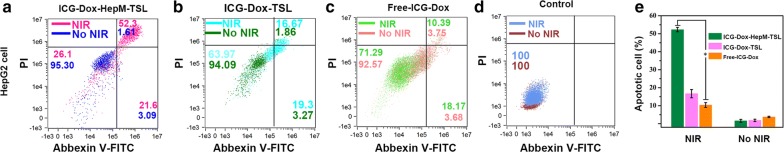
In vitro therapeutic effect. **a**–**d** The flow cytometry results of HepG2 cells. HepG2 cells were stained with an Annex V-FITC/PI apoptosis kit after incubation with ICG-Dox-**HepM-TSL**, ICG-Dox-TSL nanoparticles, free-ICG-DOX, PBSfor 4 h. **e** Apoptotic cell rate of HepG2 cells in (**a**–**d**). The concentration of Dox in ICG-Dox-**HepM-TSL**, ICG-Dox-TSL nanoparticles, free-ICG-DOX were kept the same

In order to further verify the therapeutic effect of ICG-Dox-**HepM-TSL** in the treatment of cancer in vitro, HepG2 cells were incubated with ICG-Dox-**HepM-TSL** ICG-Dox-TSL, free ICG-Dox, PBS in presence or absence of the near-infrared laser irradiation after 4 h. Then the HepG2 cells were stained with Annexin V-FITC/PI and subjected to flow cytometry analysis (Fig. 4). ICG-Dox-**HepM-TSL** strongly induced apoptosis of the HepG2 cells after exposure to near-infrared laser and the apoptosis rate of HepG2 cells was up to 52.3%, (Fig. 4a, upper right quadrant, annexin V+/PI+), while the HepG2 cells treated with ICG-Dox-**HepM-TSL** without laser irradiation or subjected to ICG-Dox-TSL were hardly in the late apoptotic stage. (Figure 4a, b, upper right quadrant). These above results indicate that ICG-Dox-**HepM-TSL** has excellent targeting effect on HepG2 cells, and under the illumination of near-infrared laser, both ICG and Dox could be released controllably, resulting in excellent in vitro therapeutic effect.

### In vivo tumor image and antitumor effect

**Fig. 5:**
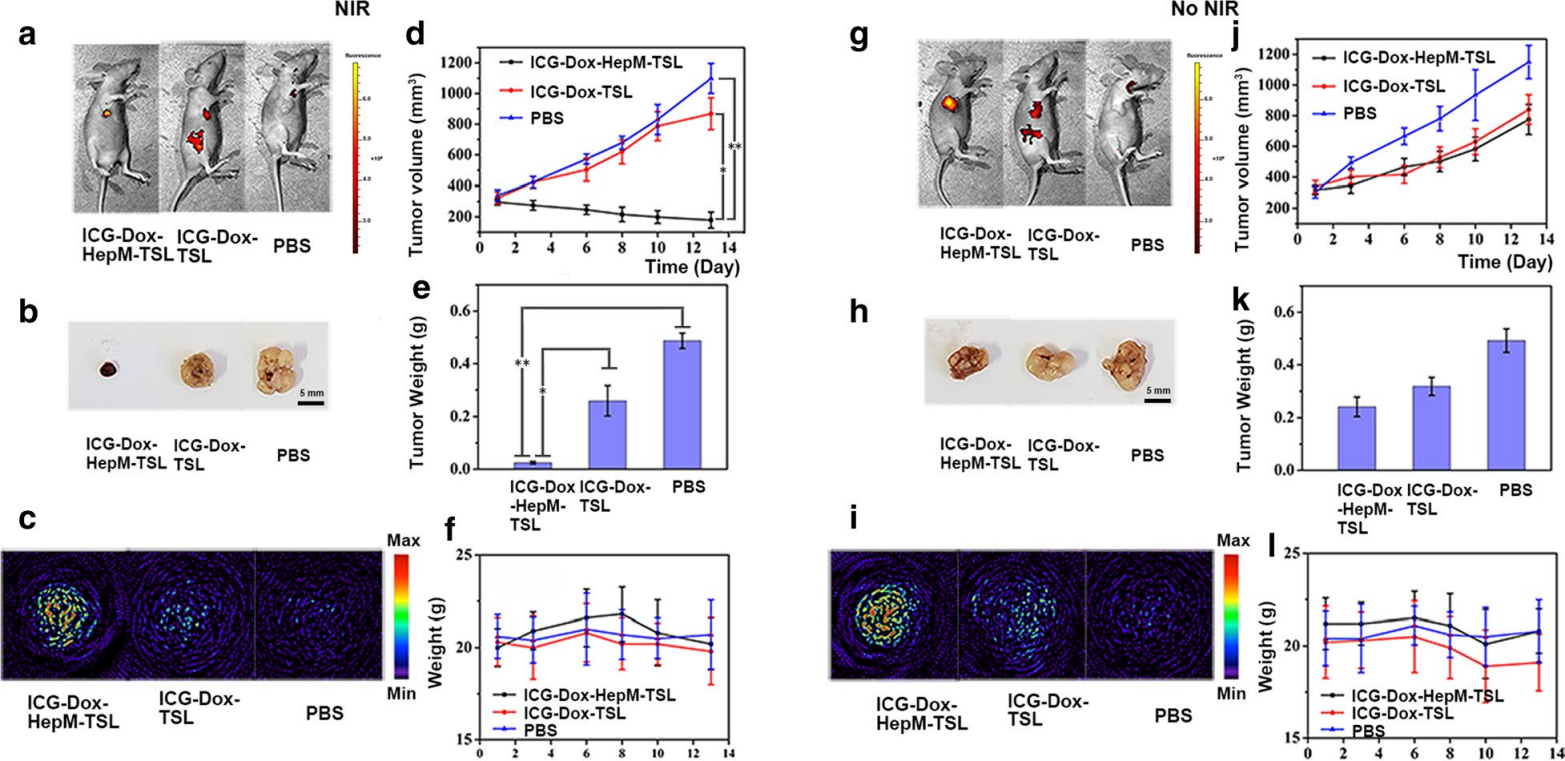
In vivo tumor image and antitumor effect. **a**–**f** Acquired from the HepG2 tumor-bearing nude mice that were intravenously injected with ICG-Dox-**HepM-TSL**, ICG-Dox-TSL and PBS under NIR irradiation (808 nm, 1 W cm^−2^). **a** Fluorescence image of HepG2 tumor-bearing nude mice 13 days after the intravenous injection of ICG-Dox-**HepM-TSL** and its counterparts. **b** Photos of the tumors extracted from the nude mice in (**a**). **c** Photoacoustic imaging of tumor sites in HepG2 tumor-bearing nude mice in (**a**). **d** Tumor weights of the nude mice after therapy. **e** Quantitative results of the HepG2 tumor relative volumes during therapy. **f** Body weights of the nude mice during therapy. **g**–**l** The corresponding data of (**a**–**f**) and acquired from the HepG2 tumor-bearing nude mice that were intravenously injected with ICG-Dox-**HepM-TSL**, ICG-Dox-TSL and PBS without of NIR irradiation

**Fig. 6:**
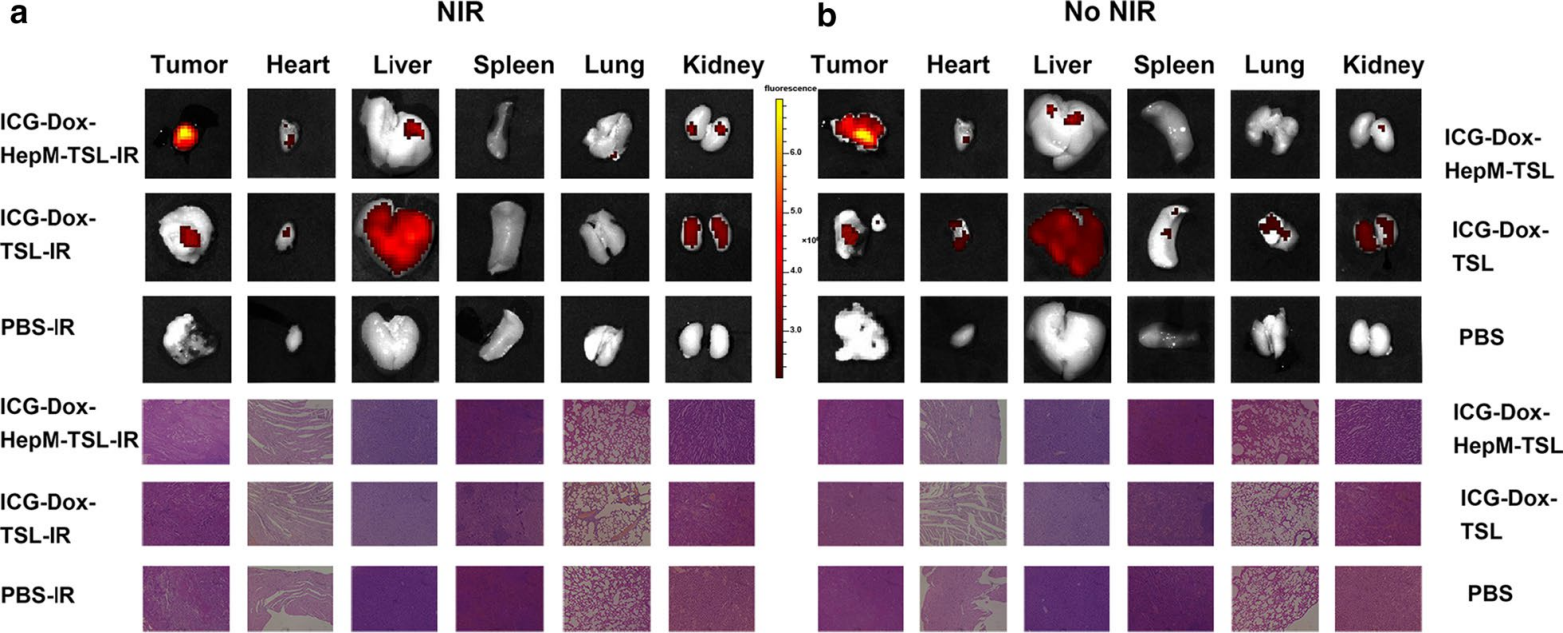
In vitro fluorescence images and H&E staining analysis of the major organs and tumors tissues extracted from the nude mice bearing the recurrent HepG2 tumor 13 days after the intravenous injection of ICG-Dox-**HepM-TSL** and its counterparts under NIR irradiation (808 nm, 1 W cm^−2^) (**a**) and without of NIR irradiation (**b**)

The in vivo experiments further proved that targeting and combination therapy of ICG-Dox-**HepM-TSL** were prominent (Fig. 5, Additional file 1: Fig. S5). The accumulation of ICG-Dox-**HepM-TSL** in the nude mice tumor sites bearing recurrent HepG2 tumor was investigated by fluorescence imaging of Dox and photoacoustic imaging 13 days after the intravenous injection with or without of NIR irradiation. The fluorescence intensity of Dox and photoacoustic intensity of ICG in the tumor region of mice treated with ICG-Dox-**HepM-TSL** under NIR irradiation were much stronger than those mice in other groups, proving the effective target ability and enrichment effect of ICG-Dox-**HepM-TSL** (Fig. 5a, b, g, h). The visual images of the extracted tumors and the tumor weight histograms showed the accurate photothermal-chemotherapy therapeutic effect of ICG-Dox-**HepM-TSL** on the recurrent tumor (Fig. 5c, d, i, j). Moreover, the volume of the recurrent tumor in the nude mice treated with ICG-Dox-**HepM-TSL** under irradiation decreased approximately 70%, while obvious increase was observed in other groups (Fig. 5e–k). Further study on the damage of organs is shown in Fig. 6. Fluorescence imaging and H&E staining analysis of the main internal organs and tumor tissues of mice showed that the internal organs of mice in the ICG-Dox-**HepM-TSL** group were less damaged.

**Fig. 7:**
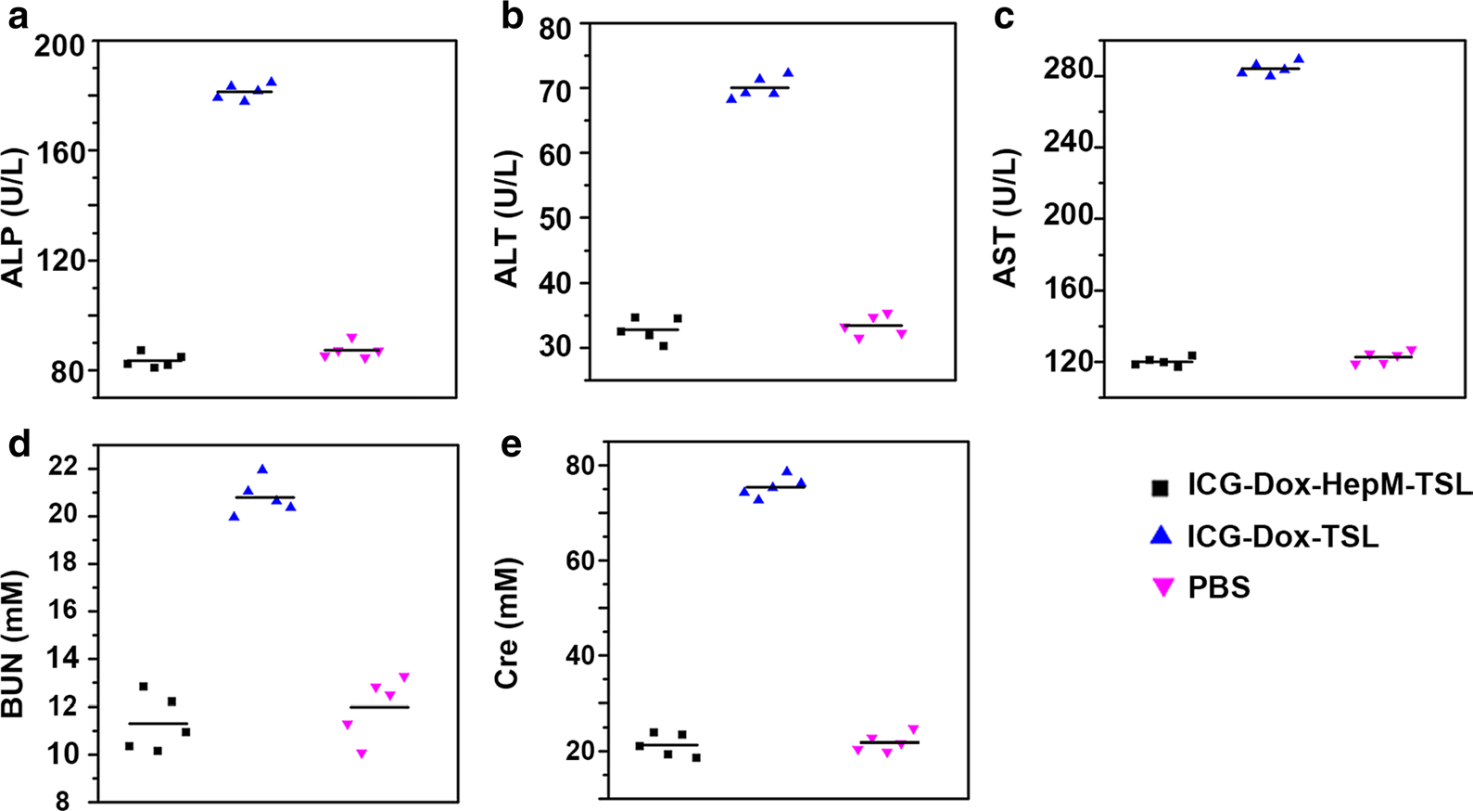
Blood biochemistry data including liver-function markers: **a** ALP, **b** ALT, **c** AST, and kidney-function markers: **d** BUN, and **e** Cre. The levels in serum collected from recurrent HepG2 tumor-bearing nude mice after therapy under NIR irradiation (808 nm, 1 W cm^−2^)

Hemocompatibility was examined by incubation erythrocytes with ICG-Dox-**HepM-TSL**, ICG-Dox-TSL, ICG, Dox and Tween 80 at gradient concentrations (Additional file 1: Fig. S6). Results indicated that, compared to Tween 80 controls (a commercial excipient intended for injectable use), ICG-Dox-**HepM-TSL** exhibited minimal hemolysis across all tested concentrations and had better hemocompatibility. During the clinical progression of chemotherapy drugs, the main side effects are due to their cumulative and dose-dependent hepatotoxicity and nephrotoxicity. Harsh renal side-effects significantly increased levels of blood urea nitrogen (BUN) and creatinine (Cre). The increase levels of alanine aminotransfease (ALT), aspartate transaminase (AST) and alkaline phosphatase (ALP) indicates serious hepatotoxicity. Blood biochemical indexs of ALT, AST, ALT, BUN, and Cre in plasma taken from recurrence-tumor mice were measured 24 h after the last injection (Fig. 7). We found that there are no significant differences between the ICG-Dox**HepM-TSL** groups and the PBS control group in the blood biochemistry indexes (ALT, AST, ALP, BUN, Cre). Together, ICG-Dox-**HepM-TSL** was characterized of low toxicity, excellent biocompatibility and satisfactory therapeutic efficiency.

## Conclusions

In summary, according to the homotypic aggregation and immune escape of cancer cells, we designed a nano-biomimetic photothermal-controlled drug-loading platform ICG-Dox-**HepM-TSL**, in which the HCC cell membrane cloaked thermosensitive liposome acted as the shell and the photothermal agent (ICG) and the chemotherapy drug (Dox) were cargoes. ICG-Dox-**HepM-TSL** could target the recurrent tumor area with the help of the homotypic HCC cell membrane. Once excited with infrared laser, ICG would generate heat; meanwhile Dox was released in a controlled manner, resulting in the synergistic effect of photothermal and chemotherapy on the recurrent HCC with little damage to normal tissues. It shows that **HepM-TSL** serves as a robust nanoplatform for recurrent HCC and provide a new strategy to the design of drug delivery platform for the treatment of cancer recurrence.

## Supplementary information

**Additional file 1: Figure S1.** TEM image of HepG2 cell membrane(a) and the ruptured liposome after the laser irradiated (b). DLS of the TSL (c), **HepM-TSL** (d), HepG2 cell membrane vesicles (e). **Figure S2.** (A) to (E) Targeting ability of **HepM-TSL** to HepG2 cells, BGC-823 cells, HeLa cells, and MCF-7 cell verified by flow cytometry. (F) Quantitative fluorescence intensities of results in (A) to (E). (G) Fluorescence images of HepG2 cells, BGC-823 cells, HeLa cells, and MCF-7 cell obtained with flow cytometry. **Figure S3.** The targeting ability of **HepM-TSL** to HepG2 cells and other cancer cells. (A) Fluorescence images of HepG2 cells, BGC-823 cells, HeLa cells and MCF-7 cells incubated with ICG-Dox-**HepM-TSL** nanoparticles. (B) Quantitative histograms of the Dox fluorescence intensities in (A). (C) Quantitative histograms of the ICG fluorescence intensities in (A). Scale bar: 75 μm. **Figure S4.** Dox and ICG loading content standard curve Dox concentration of 41.32 μg/mL. ICG concentration of 34.83 μg/mg. **Figure S5.** Schematic diagram of in vivo experiments. **Figure S6.** Hemolysis effect of the ICG-Dox-**HepM-TSL**, ICG-Dox-TSL, ICG, Dox and Tween 80.

## Data Availability

All data generated or analysed during this study are included in this published article [and its Additional file 1].

## Abbreviations

TSL: Thermosensitive liposome
Dox: Doxorubicin
ICG: Indocyanine green
HepM: Homotypic HepG2 cell membrane
ICG-Dox-**HepM-TSL**: Homotypic HepG2 cell membrane-cloaked thermosensitive liposome nanocarrier loaded ICG and Dox
TEM: Transmission electronic microscopy
HCC: Hepatocellular carcinoma
RH: Repeat hepatectomy
RFA: Radiofrequency ablation
TACE: Transarterial chemoembolization
WB: Western blot
NIR: Near-infrared fluorescent
MTT: 3-(4,5-dimethylthiazol-2-yl)-2,5-diphenyl tetrazolium bromide
DMEM: Dulbecco’s modified eagle’s medium
ALP: Alkaline phosphatase
ALT: Alanine aminotransferase
AST: Aspartate aminotransferase
BUN: Blood urea nitrogen
Cre: Serum creatinine
PBS: Phosphate buffer saline
DLS: Dynamic light scattering
COXIV: Cytochrome c oxidase IV antibody
CLSM: Confocal laser scanning microscope
