# Supplementary material for: Homotypic cell membrane-cloaked biomimetic nanocarrier for the accurate photothermal-chemotherapy treatment of recurrent hepatocellular carcinoma
Source: J Nanobiotechnology. 2020 Apr 16;18:60. doi: 10.1186/s12951-020-00617-2 (PMC7164213; doi:10.1186/s12951-020-00617-2)
Supplement: Supplementary file 1 — Additional file 1: Figure S1. TEM image of HepG2 cell membrane(a) and the ruptured liposome after the laser irradiated (b). DLS of the TSL (c), HepM-TSL (d), HepG2 cell membrane vesicles (e). Figure S2. (A) to (E) Targeting ability of HepM-TSL to HepG2 cells, BGC-823 cells, HeLa cells, and MCF-7 cell verified by flow cytometry. (F) Quantitative fluorescence intensities of results in (A) to (E). (G) Fluorescence images of HepG2 cells, BGC-823 cells, HeLa cells, and MCF-7 cell obtained with flow cytometry. Figure S3. The targeting ability of HepM-TSL to HepG2 cells and other cancer cells. (A) Fluorescence images of HepG2 cells, BGC-823 cells, HeLa cells and MCF-7 cells incubated with ICG-Dox-HepM-TSL nanoparticles. (B) Quantitative histograms of the Dox fluorescence intensities in (A). (C) Quantitative histograms of the ICG fluorescence intensities in (A). Scale bar: 75 μm. Figure S4. Dox and ICG loading content standard curve Dox concentration of 41.32 μg/mL. ICG concentration of 34.83 μg/mg. Figure S5. Schematic diagram of in vivo experiments. Figure S6. Hemolysis effect of the ICG-Dox-HepM-TSL, ICG-Dox-TSL, ICG, Dox and Tween 80. [file 12951_2020_617_MOESM1_ESM.doc]

**Additional file 1**

**Homotypic cell membrane-cloaked biomimetic nanocarrier for the accurate photothermal-chemotherapy treatment of recurrent hepatocellular carcinoma**

Yingxue Sun^1,2,†^, Wenhui Zhai^1,†^, Xiaojun Liu^1,*^, Xiangyi Song^1^, Xiaonan Gao^1^, Kehua Xu^1,*^, and Bo Tang^1,*^
^1^ College of Chemistry, Chemical Engineering and Materials Science, Key Laboratory of Molecular and Nano Probes, Ministry of Education, Collaborative Innovation Center of Functionalized Probes for Chemical Imaging in Universities of Shandong, Institute of Molecular and Nano Science, Shandong Normal University, Jinan 250014, P. R. China. Tel: 86-531-8618-2544; Fax: 86-531-8618-6033 (8618-0304)

^2^ College of Geography and Environment, Shandong Normal University, Jinan 250014, P. R. China.

^†^ These authors contributed equally.

*Corresponding author.

E-mail: tangb@sdnu.edu.cn; xukehua@sdnu.edu.cn; xiaojunliu@sdnu.edu.cn

**Contents:**

1. Materials and instruments

2. Experimental section

3. TEM image of HepG2 cell membrane

4. The targeting ability of ICG-Dox-HepM-TSL toward HepG2 cells and other cancer cells

5. Dox loading content standard curve

6. Hemolysis effect

**1. Materials and instruments**

Thermosensitive liposomes composed of dimyristoyl phosphatidylcholine (DMPC) and dipalmitoyl phosphatidylcholine (DPPC) was purchased from Xian Ruixi Biochemical Technology Co., Ltd. 3-(4,5-dimethyl-2-thiazolyl)-2,5-diphenyl-2-H-tetrazolium bromide (MTT) was purchased from Shanghai Aladdin Biochemical Technology Co., Ltd. Solvents and regents were all of analytical grade and purchased from China National Pharmaceutical Group Corp. (Shanghai, China). All solutions were prepared using ultrapure water (18.2 MΩ·cm, Millipore). The pH buffer buffer (PBS, pH 3−9) were prepared using 0.1 M citric acid and 0.2 M disodium hydrogen phosphate adjusted with 2 M NaOH or HCl solutions. The human hepatocellular liver carcinoma cell line HepG2 cells, normal liver cell line L02 cells, human gastric carcinoma cells (BGC-823 cells), cervical carcinoma cells (HeLa cells) and breast cancer cells (MCF-7 cells) were obtained from the Committee on Type Culture Collection of the Chinese Academy of Sciences. Annexin V-FITC/PI was purchased from Beijing jiamei new biological technology Co., Ltd.

Transmission electron microscopy (TEM) characterization were taken on a JEOL transmission electron microscope (JEM-2100, Shimadzu, Japan) operated at 200 kV. UV-vis absorption spectra were measured on a UV-visible spectrophotometer (UV-1700 Pharmaspec, Shimadzu, Japan). Dynamic light scattering (DLS) and zeta potential measurements were carried out on Malvern Zetasizer Nano-ZS90. Gel electrophoresis analysis and western bloot analysis were done by ChemiDocTM Touch Imaging system (Bio-Rad, Hercules, CA, USA). All pH measurements were performed with a pH-3c digital pH-meter (Shanghai LeiCi Device Works, Shanghai, China) with a combined glass-calomel electrode. Confocal fluorescence imaging was performed with a TCS SP8 confocal laser scanning microscopy (CLSM, Leica Co., Ltd. Germany) with an objective lens (×63). In vivo fluorescence imaging was conducted on the in vivo imaging system (IVIS, PerkinElmer). Absorbance in the MTT assay was measured with the microplate reader (RT 6000, Rayto, USA). The flow cytometry data were obtained using image-StreamX multispectral imaging flow cytometer (Amnis Corporation). The PA signals were recorded using an Endra Nexus128 PA tomography system (AnnArbor, Michigan).

**2. Experimental section**

**2.1 reparation of HCC cell membranes.** HepG2 cell were seeded in cell culture dishes and incubated with Dulbecco's modified Eagle's medium (DMEM) containing 10% FBS and 1% antibiotics (penicillin-streptomycin). The cells were detached with trypsin when the HepG2 cells were covered, and the cells were isolated by centrifugation at 800 rpm for 5 min, and then the cells were washed with PBS. The collected cells were resuspended in 1 mL of RIPA lysate containing 1% phenylmethanesulfonyl fluoride (PMSF) for 30 min at 4 °C. Then, the supernatant solution was collected after centrifugation at 14000 rpm for 10 min at 4 °C. To collect the cell membrane vesicles, the supernatant was subjected to further centrifugation at 20000 rpm for 60 min. The cell membrane vesicles were resuspended in PBS and saved at -80 °C.

**2.2 TEM characterization.** Negative staining (1% phosphotungstic acid) for TEM was used to characterize the bare TSL nanoparticles and the cell membrane-cloaked TSL nanoparticles and HepG2 cell membrane. The nanoparticles were dropped on a copper grid for 5 min, and then another sample was negatively stained with 2 drops of 1 wt% phosphotungstic acid. After the solution was completely dry, TEM characterization was carried out on a JEM-2100.

**2.3 Gel electrophoresis analysis and western blot analysis.** HepG2 cell lysates were added to SDS loading buffer in a 4:1 ratio and then heated at 95-100 °C for 5 min for protein denaturation, and HepG2 cell membrane protein extraction solution was processed in the same way for protein denaturation. 20 mL of protein sample and protein marker were sequentially and slowly added to the loading wells of an SDS-PAGE gel containing 12% separation gel and 5% spacer gel, the voltage was then set to 80 V for electrophoresis in the electrophoresis apparatus, and finally the voltage was changed to 120 V until the sample reached the spacer gel. The proteins in the resulting polyacrylamide gel were stained with Coomassie brilliant blue stain. The gel was imaged by the ChemiDoc^TM^ Touch Imaging System (Bio-Rad, Hercules, CA, USA).

The proteins in the resulting polyacrylamide gel were then transferred to a PVDF membrane (PerkinElmer) at 100 V for 60 min, while the Trans-Blot system was placed in ice water. The obtained PVDF membrane was blocked with 5% nonfat milk powder at room temperature for 60 min. Then, the PVDF membranes were incubated with primary antibodies for 12 h at 4 °C. The primary antibodies included antibodies to glyceraldehyde 3-phosphate dehydrogenase, galectin-1, galectin-3, CD47, cytochrome c oxidase and histone H3 (nuclear marker). After 12 h, the PVDF membrane was rewarmed at 25 °C and then washed with TBST three times, incubated with goat anti-mouse lgG HRP conjugate (1:5000 dilution in 5% nonfat milk powder) for 60 min, and then washed thrice with TBST. Finally, ECL chemiluminescent coloring solution was added dropwise, and the PVDF membranes were imaged by ChemiDoc^TM^ Touch Imaging system (Bio-Rad, Hercules, CA, USA).

**2.4 Validating the homologous targeting property of ICG-Dox-HepM-TSL with flow cytometry.**

HepG2 cells or L02 cells were seeded and cultured for 18 h in 2 mL of DMEM with 10% FBS. The supernatant was discarded, HepG2 cells and L02 cells were incubated with the the incubation buffer containing ICG-Dox-**HepM-TSL** or ICG-Dox-TSL for 4 h. To prepare the incubation buffer, 24.2 μL of ICG-Dox-**HepM-TSL** was mixed with 975.8 μL of DMEM containing 10% FBS. The incubation buffer was discarded, and the cells were washed with PBS (pH 7.4) for 3 times. Then, the cells were imaged immediately using a confocal microscope with an objective lens (× 63). Excitation of the probe-treated cells at 488 nm was performed using an argon laser, and the emitted light was collected with a META detector between 520 and 550 nm (Dox), and at 633 nm was performed using an argon laser, and the emitted light was collected with a META detector between 650 and 750 nm(ICG). The relative fluorescence intensity was measured by Zen software.

HepG2 cells, BGC-823 cells, HeLa cells and MCF-7 cells were seeded in a confocal cell culture dish and cultured for 24 h in 2 mL of DMEM with 10% FBS. After the supernatant was discarded, the HepG2 cells, BGC-823 cells, HeLa cells and MCF-7 cells were incubated with ICG-Dox-**HepM-TSL**. To prepare the incubation buffer, 24.2 μL of ICG-Dox-**HepM-TSL** was mixed with 975.8 μL of DMEM containing 10% FBS. The concentration of Dox in the final incubation solution was 5 μg/mL. The concentration of Dox in ICG-Dox-**HepM-TSL**, ICG-Dox-TSL nanoparticles, free-ICG-DOX was kept the same. The HepG2 cells and L02 cells were incubated with 200 μL of the incubation buffer in every well for 4 h. After the incubation buffer was discarded, the cells were trypsinized, collected by centrifugation at 1000 rpm for 2 min and washed thrice with PBS. Finally, the above HepG2 cells, BGC-823 cells, HeLa cells and MCF-7 cells were resuspended in 100 μL of PBS and the flow cytometry was used to obtain the data. All flow cytometry studies were conducted on an Image-StreamX multispectral imaging flow cytometer, and the data were analyzed using IDEAS software.

**2.5 In vitro cytotoxicity assay.** The cytotoxicity of ICG-Dox-**HepM-TSL** was examined by the MTT assay. HepG2 cells were seeded in 96-well microtiter plates and cultured for 24 h in 200 μL of Dulbecco’s modified Eagle’s medium (DMEM) with 10% FBS. After the supernatant was discarded, the HepG2 cells were incubated with ICG-Dox-**HepM-TSL**, ICG-Dox-TSL nanoparticles, free ICG-DOX and PBS, with or without NIR, respectively. To prepare the incubation buffer, 24.2 μL of ICG-Dox-**HepM-TSL**, ICG-Dox-TSL nanoparticles, free ICG-DOX and PBS was mixed with 975.8 μL of DMEM containing 10% FBS. The concentration of Dox in the final incubation solution was 5 μg/mL. The concentration of Dox in ICG-Dox-**HepM-TSL**, ICG-Dox-TSL nanoparticles, free-ICG-DOX was kept the same. The HepG2 cells were incubated with 200 μL of incubation buffer in every well for 4 h and after irradiation with or without NIR light. Then the incubation buffer was discarded, 100 μL of MTT solutions (5 mg/mL in PBS) were added to each well away from light. After coincubation for 4 h, the remaining MTT solution was removed, and 100 μL of DMSO was added to each well to dissolve the formed formazan crystals. The absorbance was measured at 490 nm with an ELIASA microplate reader. The experiment was repeated three times, and the data are shown as the mean± SD. The statistical data were analyzed using SPSS Statistics software, for deriving standard deviation, one-way ANOVA test and Bonferroni test. A p-value of 0.05 was taken as the level of significance and the data were labeled with (*) for P < 0.05, and for (**) for P < 0.01, Each experiment was conducted in triplicate (n=3).

**3.** **TEM image of HepG2 cell membrane**


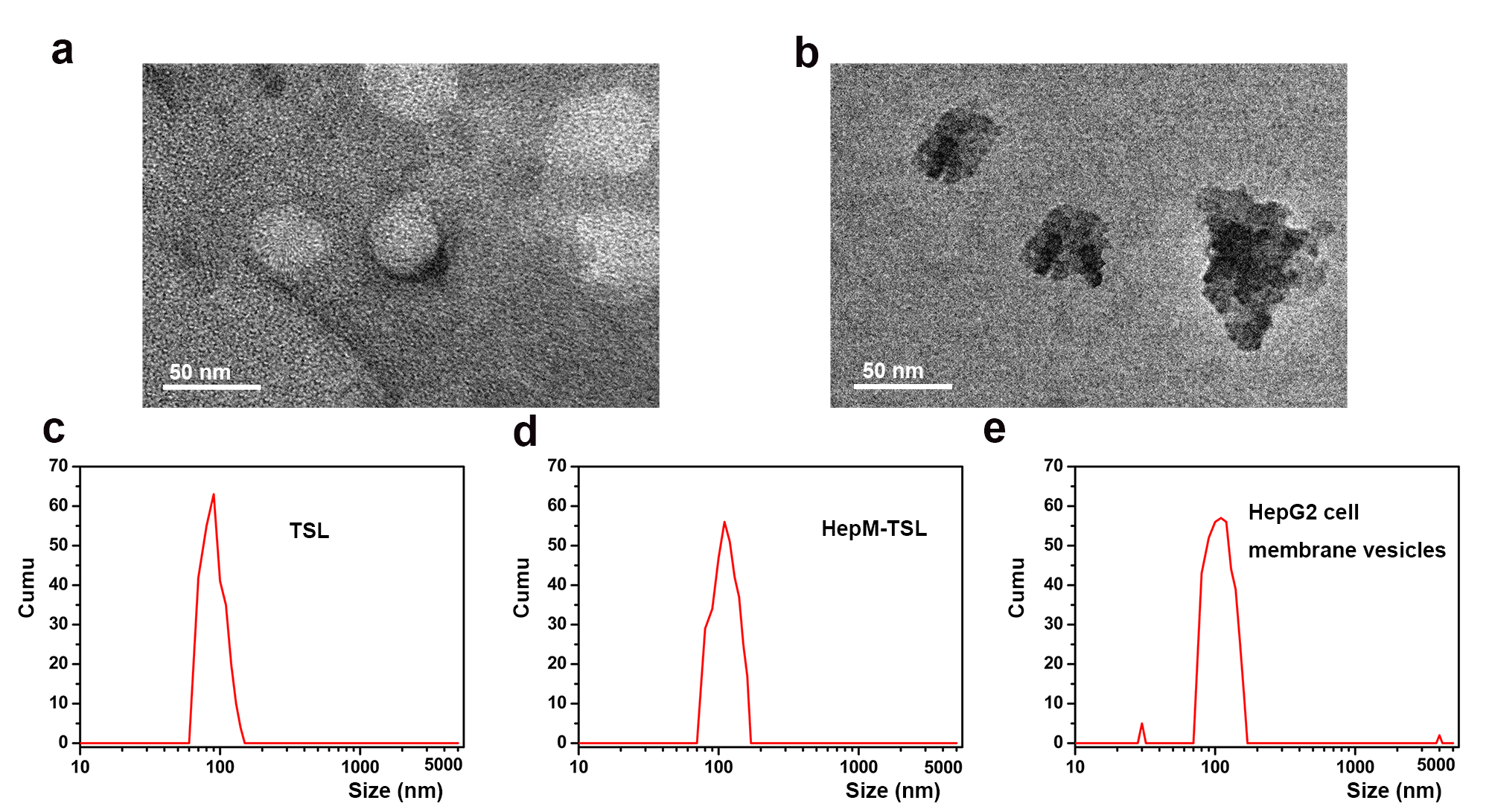


**Figure S1.** TEM image of HepG2 cell membrane(a) and the ruptured liposome after the laser irradiated (b). DLS of the TSL (c), HepM-TSL (d), HepG2 cell membrane vesicles (e).

**4.** **The targeting ability of ICG-Dox-HepM-TSL toward HepG2 cells and other cancer cells**


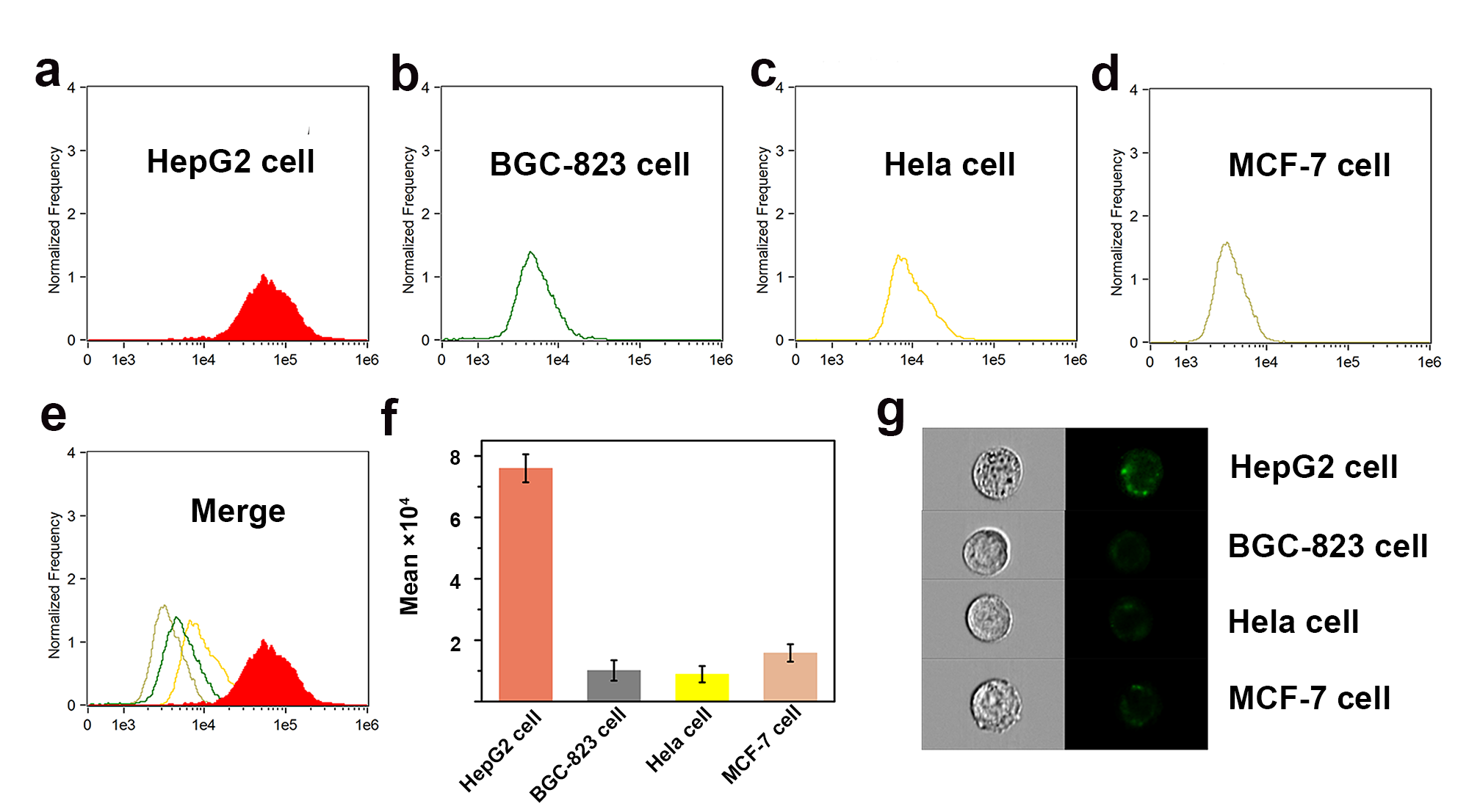


**Figure S2.** (A) to (E) Targeting ability of **HepM-TSL** to HepG2 cells, BGC-823 cells, HeLa cells, and MCF-7 cell verified by flow cytometry. (F) Quantitative fluorescence intensities of results in (A) to (E). (G) Fluorescence images of HepG2 cells, BGC-823 cells, HeLa cells, and MCF-7 cell obtained with flow cytometry.


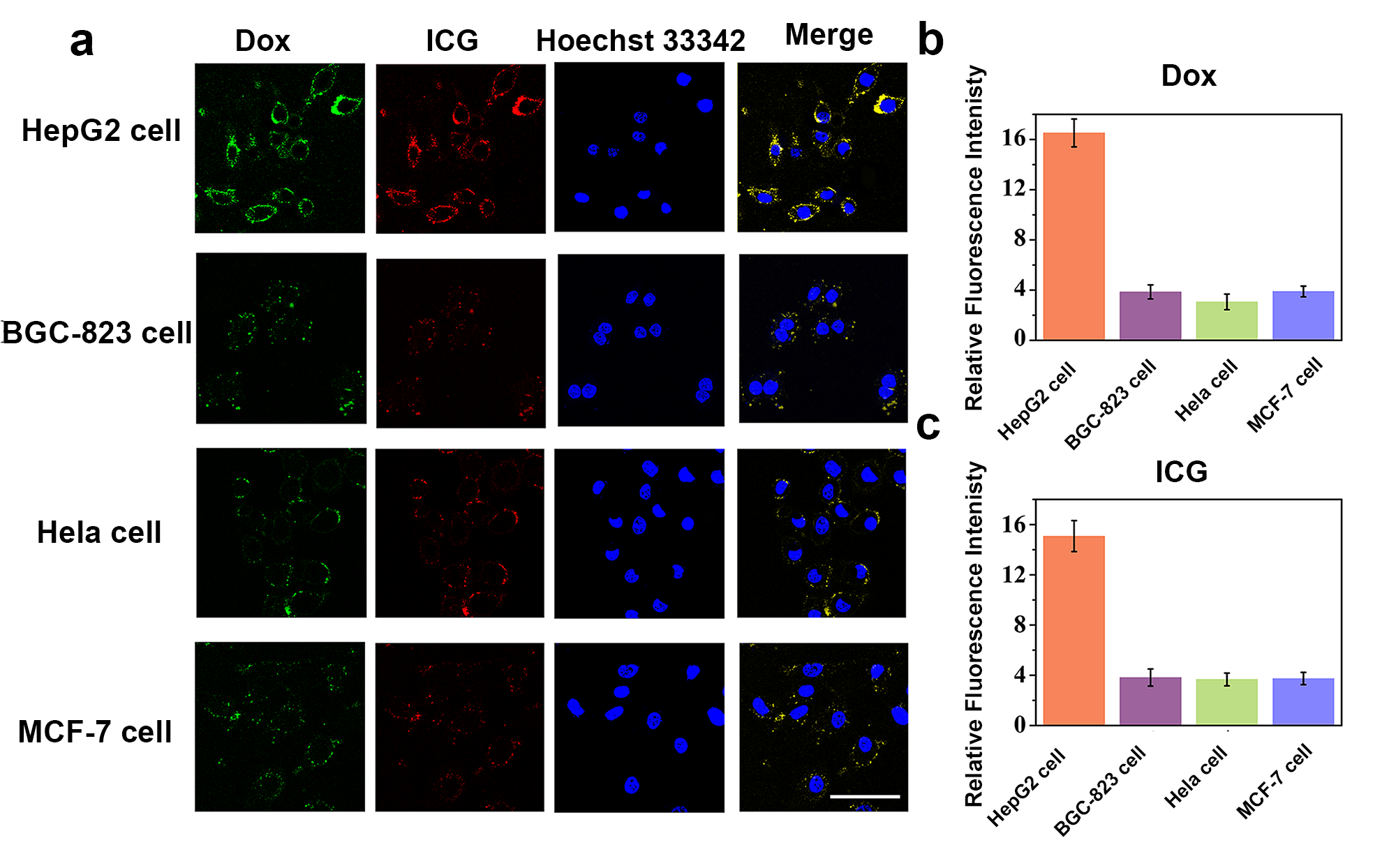


**Figure S3.** The targeting ability of **HepM-TSL** to HepG2 cells and other cancer cells. (A) Fluorescence images of HepG2 cells, BGC-823 cells, HeLa cells and MCF-7 cells incubated with ICG-Dox-**HepM-TSL** nanoparticles. (B) Quantitative histograms of the Dox fluorescence intensities in (A). (C) Quantitative histograms of the ICG fluorescence intensities in (A). Scale bar: 75 μm.

**5. Dox loading content standard curve**


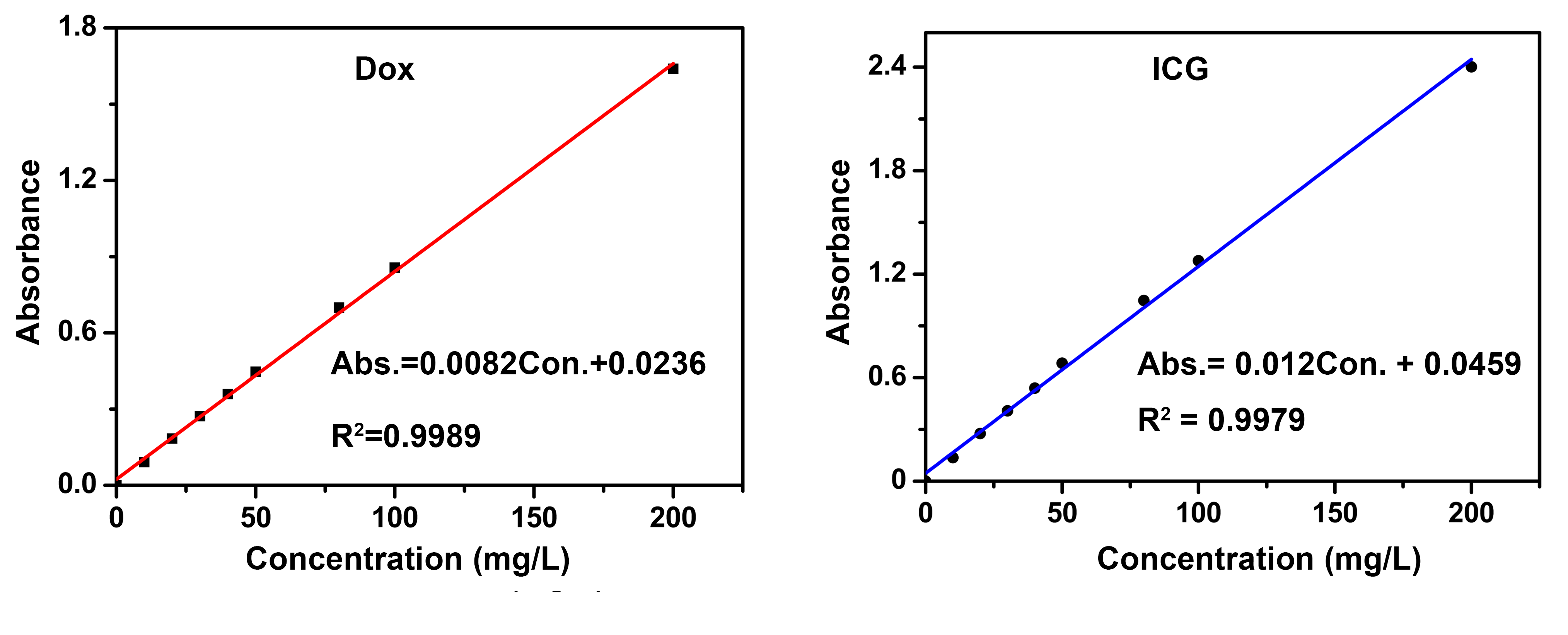


**Figure S4** Dox and ICG loading content standard curve Dox concentration of 41.32 μg/mL. ICG concentration of 34.83 μg/mg.

**6. Schematic diagram of in vivo experiments**

**
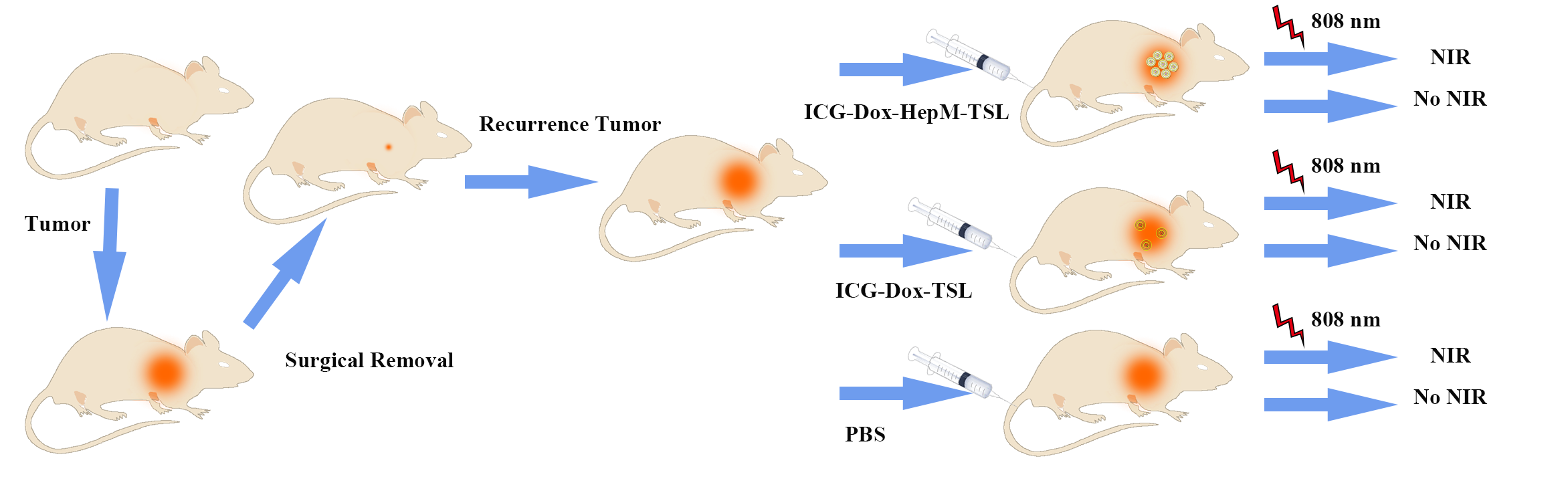
**

**Figure S5** Schematic diagram of in vivo experiments.

**7.** **Hemolysis effect**


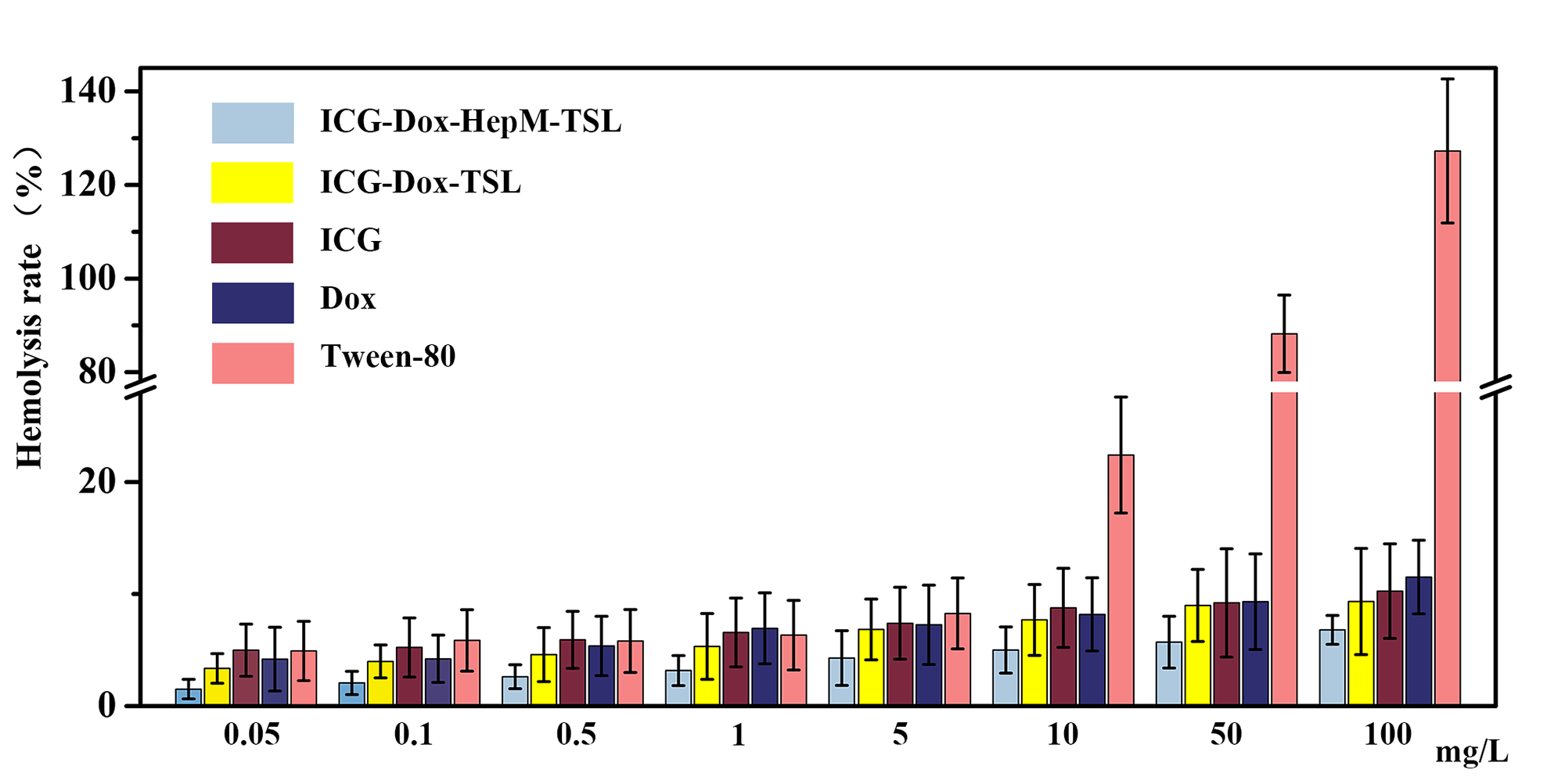


**Figure S6** Hemolysis effect of the ICG-Dox-HepM-TSL, ICG-Dox-TSL, ICG, Dox and Tween 80.
